# Leydig cell tumor in a boy with undiagnosed precocious puberty due to congenital adrenal hyperplasia

**DOI:** 10.1186/1687-9856-2015-S1-P46

**Published:** 2015-04-28

**Authors:** Dong Trieu Phuong Tran, Tan Thi Minh Nguyen, Thuy Thi Diem Hoang

**Affiliations:** 1 Department of Nephrology and Endocrinology, Children’s Hospital 2, HoChiMinh City, Vietnam; 2 Department of Pediatrics, Faculty of Medicine, Pham Ngoc Thach University of Medicine, HoChiMinh City, Vietnam

## Aims

To describe the clinical presentation and sequelae of undiagnosed congenital adrenal hyperplasia (CAH).

## Methods

Case report.

## Results

A 5-year-old boy presented with penis and testicular enlargement for 1 year. The past medical history had no severe vomiting or failure to thrive. His height was 120 cm (> 95th percentile). His penis was 7 cm and asymmetric testicles. Scrotal ultrasound detected his left testicle was 1.5×0.9 cm and his right testicle was 3.0 x 2.0 cm with a heterogeneous hypoechoic mass 1.8 x 2.0 cm at inferior pole with sheath thickness, clearly margin. His bone age was 14 years. His serum 17-hydroxypregnenolone and testosterone levels were elevated to 1768 ng/dl and 694.5 ng/dl, respectively. His serum hCG was below 1.2 IU/L and DHEA-S < 0.001 mcg/ml. An absent LH response after GnRH stimulation was recorded. He was diagnosed with CAH and treated with hydrocortisone. After one month of treatment, his serum 17-hydroxypregnenolone and testosterone levels decreased to 11 ng/dl and 20.75 ng/dl, respectively. Then, he underwent an open testis biopsy for further evaluating of the mass of his right testicle. Histological examination of the testicle demonstrated large, polygonal, and eosinophilic cells with round nuclei and prominent nucleoli, which are consistent findings with Leydig cell tumors. Thereafter, the child underwent radical orchiectomy of his right testicle.

## Conclusion

Undiagnosed congenital adrenal hyperplasia can affect normal development. Universal newborn screening is recommended for congenital adrenal hyperplasia.

Written informed consent was obtained from the patient for publication of this abstract and any accompanying images. A copy of the written consent is available for review by the Editor of this journal.

